# Blended Anion Exchange Membranes for Vanadium Redox Flow Batteries

**DOI:** 10.3390/polym13162827

**Published:** 2021-08-23

**Authors:** Tae Yang Son, Kwang Seop Im, Ha Neul Jung, Sang Yong Nam

**Affiliations:** Department of Materials Engineering and Convergence Technology, Research Institute for Green Energy Convergence Technology, Gyeongsang National University, Jinju 52828, Korea; kr6620@naver.com (T.Y.S.); rhkd685@naver.com (K.S.I.); wjdgksmf8520@naver.com (H.N.J.)

**Keywords:** redox flow battery, blended anion exchange membrane, low permeability of vanadium ion

## Abstract

In this study, blended anion exchange membranes were prepared using polyphenylene oxide containing quaternary ammonium groups and polyvinylidene fluoride. A polyvinylidene fluoride with high hydrophobicity was blended in to lower the vanadium ion permeability, which increased when the hydrophilicity increased. At the same time, the dimensional stability also improved due to the excellent physical properties of polyvinylidene fluoride. Subsequently, permeation of the vanadium ions was prevented due to the positive charge of the anion exchange membrane, and thus the permeability was relatively lower than that of a commercial proton exchange membrane. Due to the above properties, the self-discharge of the blended anion exchange membrane (30.1 h for QA–PPO/PVDF(2/8)) was also lower than that of the commercial proton exchange membrane (27.9 h for Nafion), and it was confirmed that it was an applicable candidate for vanadium redox flow batteries.

## 1. Introduction

Because natural energy, e.g., solar, wind, and geothermal, are fluctuating forms of energy, the need for energy storage technologies is expanding. For this reason, it is very important to fabricate efficient energy storage devices. Among various energy storage technologies, electrochemical devices have gained a lot of interest, especially redox flow batteries (RFBs) [1,2]. These redox flow batteries are divided according to their active material into metal and organic material based batteries [3]. Especially, vanadium redox flow batteries (VRFB) are suitable for large scale power grid energy storage, as brought up for the first time in 1985 by M. Skyllas-Kazacos [4]. In addition, vanadium redox flow batteries (VRFB) have many advantages such as high power capacity, long cycle life, and flexible design [5,6,7]. 

VRFBs consist of three key components: electrode, electrolyte (active material), and membrane. Among these components, the membrane plays an important role and has a huge impact on VRFB performance. The membrane divides the positive and negative electrodes to prevent short circuit and mixing of active materials. The membrane also transfers ions to maintain charge balance. So, an ideal membrane for a VRFB system should have stable vanadium impermeability, high ion conductivity, and mechanical and chemical stability [8,9]. These days, membranes made of Nafion, invented by Dupont in the US, are the most widely used VRFB membranes in light of their excellent proton conductivity and remarkable physicochemical stability. Despite these advantages, high cost and the still high permeability for vanadium limit their commercial application [10]. For the above reasons, many researchers have turned their eyes to finding ideal membranes using hydrocarbon polymers to replace Nafion membranes. 

Hydrocarbon polymers mainly include poly(phenylene oxide) (PPO) [11,12], poly(ether ether ketone) (PEEK) [13], polyimide (PI) [14], polybenzimidazole (PBI) [15,16], poly(styrene-ethylene-butylene-styrene) (SEBS) [17,18,19], and so on. Due to the excellent mechanical and thermal stability and low vanadium permeability of hydrocarbon polymers, these materials have been extensively used in the field. 

Novel hydrocarbon anion exchange membranes (AEMs) have attracted a lot of attention for fuel cell, water electrolysis, and VRFB applications, and seem to fulfill the requirements mentioned above [20,21,22]. Among various advantages of AEM for VRFBs, one is the exclusion of vanadium ions from the membrane phase because vanadium ions carry the same charge as functional groups of AEMs (Donnan exclusion effect). As a result, differential vanadium crossover is reduced, which eventually results in high coulombic efficiency and long self-discharge duration of the VRFB [23,24,25]. Another advantage is that membrane degradation through chemical oxidation by VO^2+^ is low [26,27]. 

Because such anion exchange membranes have relatively low ion conductivity, a polymer having a high degree of substitution is required to dramatically improve it. However, due to the high degree of substitution, the dimensional stability is weakened due to excessive hydrophilicity. There are various methods of improving the dimensional stability, including methods of preparing a pore-filling membrane [11], a cross-linked membrane [12], and a blended membrane [28]. Among these, the blended membrane is a very good option. The reason is that the polymer used for blending is not only inexpensive, but also relatively simple to optimize compared to pore-filling membranes and cross-linked membranes. 

Therefore, in this study, polyvinylidene fluoride (PVDF) polymer, used in membrane bio-reactor (MBR) or membrane aeration bio-reactor (MABR) application because of excellent mechanical properties and hydrophobicity [28,29], was used and the membrane was prepared by appropriately blending polyphenylene oxide (PPO) polymer introduced with quaternary ammonium. Based on the results of the evaluation, we tried to confirm whether this material is applicable for redox flow batteries.

## 2. Materials and Methods

### 2.1. Materials

Poly (2,6–dimethyl–1,4–phenylene oxide) (PPO, poly (phenylene oxide)) was purchased from Asahi Kasei Corp (Chiyoda, Tokyo, Japan). N–bromosuccinimide (NBS), 2,2′–azobis (2–methylpropionitrile) (AIBN) and the trimethylamine solution (TMA solution) were obtained from Sigma Aldrich Chemical Corp (Burlington, MA, USA). Polyvinylidene fluoride (PVDF, Kynar^®^ 761) for blending was from ARKEMA Corp (Colombes, France). Chlorobenzene, N–methyl–2–pyrrolidone (NMP), ethyl acetate (EA), methyl alcohol (MeOH), potassium hydroxide (KOH), hydrochloric acid solution and sodium hydroxide solution were purchased from Daejung Chemical Corp (Siheung, Korea).

### 2.2. Synthesis of Brominated Poly (Phenylene Oxide) (Br–PPO)

Brominated poly (phenylene oxide) (Br–PPO) was synthesized in a 250 mL two-neck round bottom flask equipped with a reflux condenser and a nitrogen inlet. Five grams (41.614 mmol) of poly (phenylene oxide) (PPO) polymer was solubilized in 86 mL of chlorobenzene. Next, 0.05 g (0.208 mmol) of 2,2′–azobis (2–methylpropionitrile) (AIBN) was added. Then, the resulting solution was stirred for 20 min at 50 °C under nitrogen atmosphere. After the generation of radicals due to the initiator AIBN, 3.70 g (20.807 mmol) of N–bromosuccinimide (NBS) was added to the solution in the flask. The temperature was then raised to 135 °C and the reaction was continued for 3 h. The viscous solution was cooled to room temperature and then precipitated in 5 to 6 times methanol (MeOH) as non-solvent. After thorough stirring and washing with MeOH, the precipitate was fully dried in a vacuum oven at 60 °C for 24 h to yield 9.49 g of polymer (95% yield) [11].

### 2.3. Synthesis of Poly(Phenylene Oxide) Containing Quaternary Ammonium (QA–PPO)

Five grams of Br–PPO was dissolved in 95 g of N–methyl–2–pyrrolidone (NMP) solvent at 60 °C. When the TMA solution (6.24 mL) was injected, the formation of precipitate was prevented with a vortex mixer. Then, the reaction was carried out so that all of the bromine ions could be replaced with quaternary ammonium groups at 60 °C. Afterward, the resulting solution was precipitated in ethyl acetate (EA) and washed with the same non-solvent until the unreacted TMA was removed. After washing sufficiently several times, the precipitate was dried in a vacuum oven to collect the final product (QA–PPO) [11].

### 2.4. Preparation of Blended Anion Exchange Membranes (BAEMs)

Blended anion exchange membranes of QA–PPO/PVDF were prepared by the direct casting method. The casting solution was prepared by dissolving the QA–PPO/PVDF polymers in NMP solvent at ratios of 2:8, 3:7, and 4:6. Before pouring into a Petri dish to prevent phase separation, the solution was thoroughly mixed with a vortex mixer, and then poured and cast. After that, the Petri dish was dried in a vacuum oven at 60 °C for 12 h. 

The BAEMs were impregnated in DI-water to remove them from the Petri dish, and the BAEMs eventually fell off by themselves. To evaluate the properties, the BAEM thus obtained was put in a 1M KOH solution for at least 12 h; all counter ions were replaced with hydroxide ions. The hydroxide-exchanged BAEMs were washed several times with DI-water to remove any residual KOH and stored in DI-water until the characterization process.

The blended anion exchange membranes were named QA–PPO/PVDF(X/Y) membrane, where X and Y represent the weight ratios of QA–PPO and PVDF.

### 2.5. Experimental Techniques

#### 2.5.1. H–NMR

The ^1^H–NMR spectra of Br-PPO were obtained on DRX300 (300 MHz) (Bruker, Billerica, MA, USA). Chloroform–d was used as the NMR solvent.

#### 2.5.2. Ion Exchange Capacity, Water Uptake, Swelling Ratio and Hydration Number

The back-titration method was used for the anion exchange membrane. The BAEM was immersed in 0.01 M HCl solution for 24 h, and the IEC value was measured by titration using a 0.01 M NaOH solution. After finishing the titration process, the BAEM was removed and dried in a vacuum oven at 60 °C. The weight of the dried BAEM was then measured. Finally, the ion exchange capacity was calculated using the following Equation (1):

(1) $$IEC\;\left(meq/g\right)=\frac{\left[\left(V_{HCl}\times M_{HCl}\right)-\left(V_{NaOH}\times M_{NaOH}\right)\right]}{W_{dry}}$$

where *M_HCl_* (M) and *V_HCl_* (mL) are, respectively, the concentration and volume of the initial HCl solution. *M_NaOH_* (M) and *V_NaOH_* (mL) are the concentration and volume of standard NaOH solution used for titration, respectively. *W_dry_* (g) is the weight of the dry BAEM.

The water uptake was calculated according to the weight change of the BAEM. To determine the water uptake value, the BAEM was cut into an appropriate size, and then weighed (*W_dry_*). The samples were then immersed in DI water for 24 h. Excess water on the sample surface was wiped away with tissue paper and the sample weight was determined (*W_wet_*). The water uptake was calculated using the following Equation (2):

(2) $$Water\;uptake\;\left(\%\right)=\frac{\left(W_{wet}-W_{dry}\right)}{W_{dry}}\times 100$$

The hydration number of water molecules per ionic group (*λ*) was determined using the following Equation (3):

(3) $$Hydration\;number\;\left(\lambda \right)=\frac{\left(W_{wet}-W_{dry}\right)/W_{dry}}{18\times IEC}$$

Swelling ratio (%) of BAEM was measured by immersing the rectangular shaped membranes into deionized water, and the changes of size were calculated using the following Equation (4):

(4) $$Swelling\;ratio\;\left(\%\right)=\frac{\left(L_{wet}-L_{dry}\right)}{L_{dry}}\times 100$$

where *L_wet_* and *L_dry_* represent the length of the wet and dry BAEM, respectively.

#### 2.5.3. Mechanical Properties: Tensile Strength, Elongation at Break and Young’s Modulus

The mechanical properties of the blended anion exchange membrane were measured by using a universal testing machine (UTM, LR10K of LLOYD) according to the ASTM D635 6. To confirm the properties, the tension speed was set to 10 mm/min.

#### 2.5.4. Hydroxide Conductivity

The hydroxide conductivity of the BAEM is an important factor. It was determined using the measured value of the membrane resistance. Hydroxide conductivity tests of the BAEMs were carried out in a temperature range from 25 °C to 80 °C and at a relative humidity of 100 % by impedance method using electrochemical spectroscopy (SP-300, Bio Logic Science Instrument, Seyssinet-Pariset, France). Finally, the hydroxide conductivity was calculated using the following Equation (5):

(5)   σ=L/R×A

σ is the ion conductivity, *R* is the electrical resistance, and *L* and *A* are, respectively, the thickness and area of the membrane.

#### 2.5.5. Vanadium Permeability

Among the oxidation states of vanadium, VO^2+^ is usually subject to permeability measurement because it is a more robust species towards oxidation in air. BAEM samples were sandwiched between two electrolyte chambers with a volume of 100 mL. The left chamber was filled with 1 M VOSO_4_ in 2 M H_2_SO_4_, while the left chamber was filled with 1 M MgSO_4_ in 2 M H_2_SO_4_. MgSO_4_ was used to balance the ion strength and reduce the water transfer due to the osmotic effect. Magnetic stirrers were used in both chambers to avoid concentration polarization at the membrane surfaces. The concentrations of VO^2+^ in the BAEM samples were measured using a UV-vis spectrometer. The vanadium permeability can be calculated according to Fick’s diffusion law. The following Equation (6) was used to calculate the permeability (*P*, cm^2^min^−1^) of VO^2+^:

(6) $$P=\frac{LV_{R}}{\left(A(C_{L}-C_{R}\left(t\right)\right)}\frac{dC_{R\left(t\right)}}{dt}$$

where *L* is the thickness of the BAEMs, *V_R_* is the volume of the right chamber, *A* is the active area of the BAEMs, *C_L_* is the concentration of VO^2+^ in the left chamber, *C_R_*_(*t*)_ is the concentration of vanadium in the right chamber at the time of *t*, and *t* itself is the measurement time [20].

#### 2.5.6. Redox Flow Battery Measurement

The blended anion exchange membranes (BAEMs) were assembled with two graphite felt electrodes; the effective area was 9 cm^2^ (Standard Energy, Daejeon, Korea). Two 200 mL active solutions of 1.5 M V^3+^/VO^2+^ in 3 M H_2_SO_4_ were used as positive and negative electrolytes. The electrolytes were cyclically pumped into the corresponding half-cell using peristaltic pumps with a flow rate 150 mL/min. The redox flow battery was charged to 1.7 V at a current density of 100 mA/cm, and the open circuit voltage was checked by a battery test system (SP-240, Bio Logic Science Instrument, Seyssinet-Pariset, France) [30].

## 3. Results and Discussions

### 3.1. Structure Analysis

**Figure 1:**
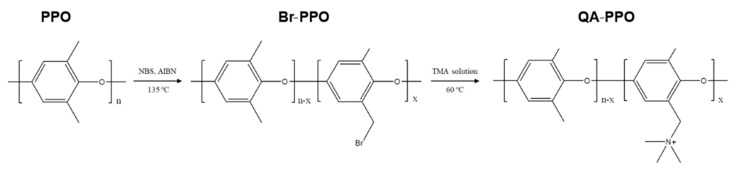
Scheme of the polyphenylene oxide containing quaternary ammonium (QA–PPO).

Figure 1 shows the preparation of the polyphenylene oxide containing quaternary ammonium (QA–PPO). The QA–PPO was synthesized by quaternization via a bromination reaction. In the case of the bromination step, PPO was subjected to a bromination reaction with N–bromosuccinimide (NBS) and AIBN as an initiator to yield Br–PPO. The Br–PPO was reacted with trimethylamine solution (TMA solution), yielding QA–PPO. The structure of the final polymer was confirmed by ^1^H–NMR spectroscopy.

**Figure 2:**
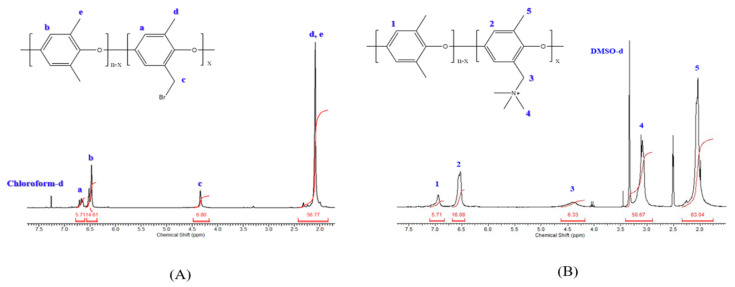
^1^H–NMR spectra of (**A**) brominated polyphenylene oxide (Br–PPO) and (**B**) polyphenylene oxide containing quaternary ammonium (QA–PPO).

Figure 2 shows the ^1^H-NMR spectra of Br-PPO and QA–PPO. In Figure 2A, the peak at 4.48 ppm was assigned to the hydrogen peak of the Br substituted portion in the methyl group of PPO (2H, –CH_2_Br). The chemical shifts at 6.60 ppm and 6.40 ppm were attributed to the hydrogen peak of the substituted benzene (1H, Ar–H) and the unsubstituted benzene (1H, Ar–H), respectively. The substitution of the quaternary ammonium group was continuously confirmed by the appearance after the reaction of trimethylamine, corresponding to the methyl group of quaternary ammonium (9H, –N(CH_3_)_3_) (Figure 2B). The complete conversion to the quaternary ammonium confirmed the chemical shift. Bromomethyl hydrogen (2H, –CH_2_Br) at 4.48 ppm completely shifted to 4.5 ppm [11,31].

### 3.2. Fabrication of Blended Anion Exchange Membranes (Baems) with Polyvinylidene Fluoride (PVDF)

**Figure 3:**
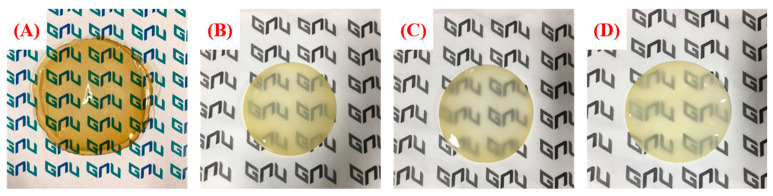
Pictures of blended anion exchange membranes: (**A**) pristine QA–PPO, (**B**) QA–PPO/PVDF(2/8), (**C**) QA–PPO/PVDF(3/7), and (**D**) QA–PPO/PVDF(4/6).

In Figure 3, photographs of the fabricated blended anion exchange membrane are compared. In the case of the pristine QA–PPO membrane not blended with PVDF, the sample is dark brown and transparent. However, it can be seen that, when PVDF is mixed, it becomes opaque, and there is no significant difference in appearance with respect to the ratio. In addition, although a thin thickness is important for high ionic properties, in a redox flow battery, a thick thickness is required because the thin thickness may increase the penetration of vanadium ions [32]. Therefore, the BAEMs were manufactured with a thickness of about 50 μm or more.

**Figure 4:**
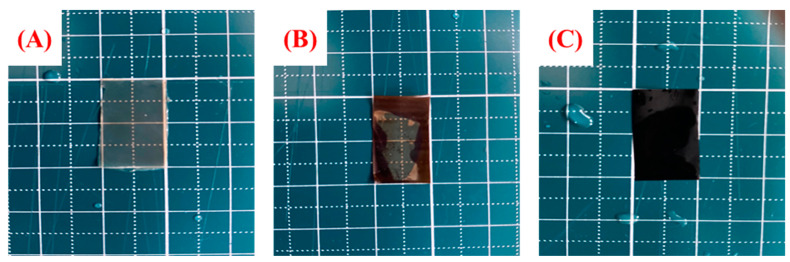
Changes in appearance of BAEMs after impregnation with 1 M KOH solution: (**A**) QA–PPO/PVDF(2/8), (**B**) QA–PPO/PVDF(3/7), and (**C**) QA–PPO/PVDF(4/6).

To proceed with the characterization of the prepared BAEMs, all counter ions were changed to hydroxide ions by immersion in 1 M KOH solution. At this time, the BAEMs were not immersed in 1 M KOH for a long time. The reason is that PVDF is defluorinated in 1 M KOH solution, so the substitution was carried out for a suitable time [33,34]. Next, Figure 4 shows the changes in appearance when BAEMs were immersed in KOH solution for a long time.

The appearance of the BAEMs changed due to defluorination; it can be expected that the excellent stability of PVDF was lost due to defluorination of PVDF [33,34]. It can be seen that the lower the PVDF content, the more severe this defluorination is; and, the lower the PVDF content, the lower the polarity difference is between the polymers, which makes the mixture better, and it is judged to be more stable due to the strong hydrogen bonding. That is, it is thought that, at the ratio of 2:8, the repulsive force between the hydrophilic polymer and the hydrophobic polymer is relatively less than that of the 4:6 ratio due to the large ratio difference between QA–PPO and PVDF. At the same time, it can be assumed that the possibility of hydrogen bond formation is higher because fluoride is more abundant in PVDF polymer.

### 3.3. Mechanical Properties

Table 1 shows the differences in the mechanical strength of the pristine membrane and the BAEMs. The existence of the PVDF increased the overall mechanical strength. In the case of the pristine membrane, when the hydrophilicity increased due to the high degree of substitution, the mechanical strength weakened because of water absorption and resulting hydration. On the other hand, when a polymer with excellent physical properties, polyvinylidene fluoride (PVDF), was introduced, the overall mechanical strength improved [28]. In detail, as the proportion of PVDF increased, the mechanical strength tended to increase. Especially, QA–PPO/PVDF(2/8) showed a tensile strength of 29.6 MPa, an elongation at break of 7.6%, and a Young’s modulus of 695.3 MPa.

### 3.4. IEC, WU, SR and λ Values

Table 2 summarizes the ionic properties of prepared BAEMs, such as IEC, WU, SR and hydration number. First, it can be seen that the ion exchange capacity decreases as the content of PVDF without ion exchange groups increases. So, it can be seen that the water uptake and the swelling ratio show similar trends. However, the hydration number shows a different trend, which can be thought of as the difference due to the more hydrogen bonds, de-scribed above.

### 3.5. Ion Conductivity (Hydroxide Conductivity)

**Figure 5:**
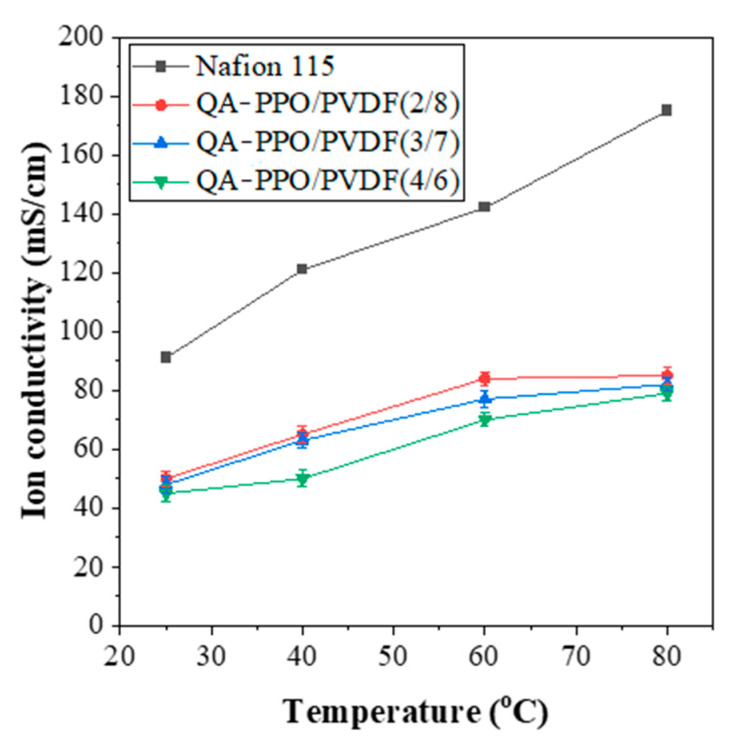
Ion conductivities of various BAEMs.

The ion conductivities of the BAEMs with different ratios of PVDF were measured at 25, 40, 60, and 80 °C. First, the conductivities tended to be proportional to the temperature, as shown in Figure 5 and Table 3. In particular, QA–PPO/PVDF (2/8) exhibited an ionic conductivity of 85 mS/cm at a temperature of 80 °C (Table 3). However, as the content of QA–PPO in-creased, the ionic conductivity did not increase. It was found that the ionic conductivity eventually showed the same trend as the hydration number. Considering the above performances of the QA–PPO/PVDF (2/8) sample, this sample was used to measure the performance of the redox flow battery.

### 3.6. Vanadium Ion Permeability

**Figure 6:**
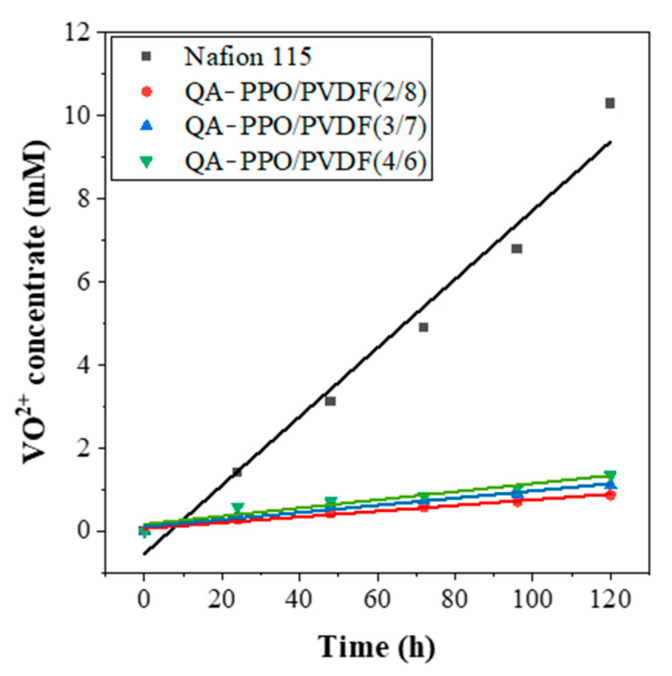
Permeability of VO^2+^ ion of the vanadium redox flow battery assembled with BAEMs.

The crossover of active materials such as vanadium species in redox flow batteries is a crucial issue that could lead to a serious self-discharge problem. In an operating battery system, the membrane is in contact with concentrated H_2_SO_4_, containing vanadium cat-ions with four different oxidation states. At the same time, due to the partitioning of ions into the membrane, the concentration electrolyte affects the ion transport behavior and ion equilibrium within the membrane [35,36]. The P value of the tested membranes was calculated by the change of VO^2+^ concentration in the MgSO_4_ solution with time, as shown in Figure 6. It can be seen that the VO^2+^ concentrations decreased as the PVDF ratio increased, which can be explained by the greater hydrophobicity as the PVDF ratio increased. When the hydrophilicity is excellent, the crossover phenomenon becomes severe [37]. However, in this case, it is judged to be the effect of reducing the crossover phenomenon by the positive charge of quaternary ammonium and the hydrophobicity of PVDF.

### 3.7. Open Circuit Voltage

**Figure 7:**
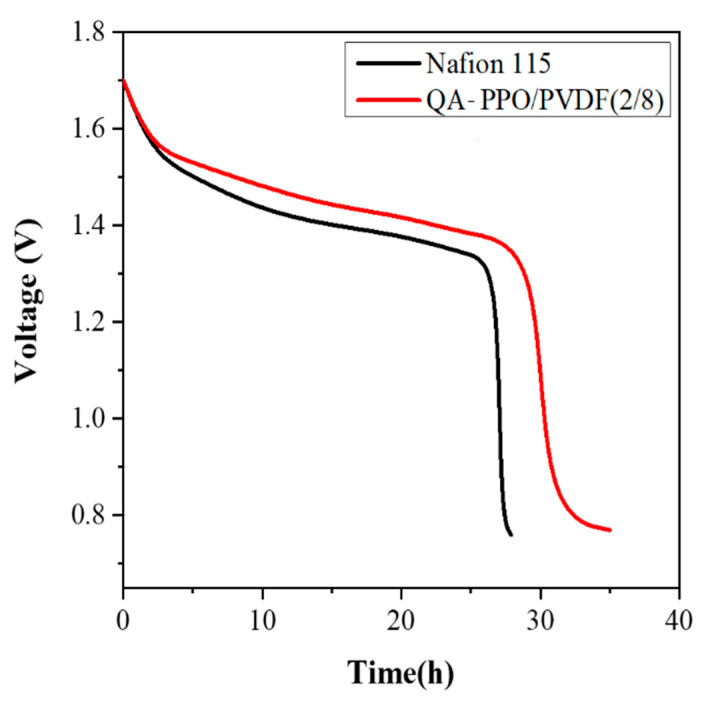
Open circuit voltage of vanadium redox flow battery assembled with Nafion 115 and QA–PPO/PVDF(2/8).

To systematically study the performance of BAEMs, the OCV of VRFB single cells was measured. The OCV is a critical parameter used to verify the vanadium ion cross rate in the membranes, as the vanadium ions crossing the membrane result in self-discharge and the cell voltage declines accordingly. Figure 7 shows that the voltage decay of cells assembled with BAEM (30.1 h for QA–PPO/PVDF(2/8)) is lower than that of the commercial membrane (27.9 h for Nafion 115). It can be seen that the BAEM, QA–PPO/PVDF(2/8), suppresses the permeation of vanadium ions, in good agreement with the results in Figure 6 [38]. Accordingly, the membrane blended using PVDF is expected to be an alternative to Nafion membrane, as it has better performance.

## 4. Conclusions

Blended anion exchange membranes with excellent mechanical properties and polyphenylene oxide containing a quaternary ammonium group (QA–PPO) were prepared by a facile method using polyvinylidene fluoride (PVDF). In particular, the QA–PPO/PVDF (2/8) sample had a relatively higher hydration number due to hydrogen bonding than did the other blended anion exchange membranes. In addition, since the prepared BAEMs had positive charges compared to commercial proton exchange membranes such as a Nafion membrane, the permeability for VO^2+^ ion was relatively low. At the same time, it was confirmed that the self-discharge of the vanadium redox flow battery was relatively lower than that with Nafion. Therefore, based on the above data, we believe that our blended anion exchange membranes are promising candidates for vanadium redox flow battery systems.

## Figures and Tables

**Table 1:** Mechanical properties of BAEMs.

Membrane	Mechanical Properties		
	Tensile Strength (MPa)	Eleongation at Break (%)	Young’s Modulus (MPa)
QA–PPO	22.5	4.2	535.7
QA–PPO/PVDF(2/8)	29.6	7.6	695.3
QA–PPO/PVDF(3/7)	25.3	9.0	665.2
QA–PPO/PVDF(4/6)	23.7	11.2	612.3

**Table 2:** The ionic parameters of BAEMs.

Sample	IEC Value (meq/g)	Water Uptake (%)	Swelling Ratio (%)	Hydration Number (λ)
QA–PPO	2.31	55.6	18.2	13.37
QA–PPO/PVDF(2/8)	0.61	11.5	3.9	10.47
QA–PPO/PVDF(3/7)	1.08	17.0	7.4	8.74
QA–PPO/PVDF(4/6)	1.75	24.5	13.8	7.78

**Table 3:** Hydroxide conductivity values of various BAEMs.

Sample	Hydroxide Conductivity (mS/cm)			
	In DI Water			
	25 °C	40 °C	60 °C	80 °C
Nafion115	91	121	142	175
QA–PPO/PVDF(2/8)	50	65	84	85
QA–PPO/PVDF(3/7)	48	63	77	82
QA–PPO/PVDF(4/6)	45	50	70	79

## Data Availability

The data supporting the findings of this manuscript are available from the corresponding authors upon reasonable request.

## References

[1] AL Shaqsi A.Z., Sopian K., Al-Hinai A. (2020). Review of Energy Storage Services, Applications, Limitations, and Benefits. Energy Rep..

[2] Sánchez-Díez E., Ventosa E., Guarnieri M., Trovò A., Flox C., Marcilla R., Soavi F., Mazur P., Aranzabe E., Ferret R. (2021). Redox Flow Batteries: Status and Perspective Towards Sustainable Stationary Energy Storage. J. Power Sources.

[3] Winsberg J., Hagemann T., Janoschka T., Hager M.D., Schubert U.S. (2017). Redox-Flow Batteries: From Metals to Organic Redox-Active Materials. Angew. Chem. Int. Ed..

[4] Kim K.J., Park M., Kim Y., Kim J.H., Dou S.X., Skyllas-Kazacos M. (2015). A Technology Review of Electrodes and Reaction Mechanisms in Vanadium Redox Flow Batteries. J. Mater. Chem. A.

[5] Cunha Á., Martins J., Rodrigues N., Brito F. (2015). Vanadium Redox Flow Batteries: A Technology Review. Int. J. Energy Res..

[6] Weber S., Peters J.F., Baumann M., Weil M. (2018). Life Cycle Assessment of a Vanadium Redox Flow Battery. Environ. Sci. Technol..

[7] Jiang H., Sun J., Wei L., Wu M., Shyy W., Zhao T. (2020). A High Power Density and Long Cycle Life Vanadium Redox Flow Battery. Energy Storage Mater..

[8] Schwenzer B., Zhang J., Kim S., Li L., Liu J., Yang Z. (2011). Membrane Development for Vanadium Redox Flow Batteries. ChemSusChem.

[9] Tang Z., Keith R., Aaron D.S., Lawton J.S., Papandrew A.P., Zawodzinski T.A. (2012). Proton Exchange Membrane Performance Characterization in VRFB. ECS Trans..

[10] Jiang B., Wu L., Yu L., Qiu X., Xi J. (2016). A Comparative Study of Nafion Series Membranes for Vanadium Redox Flow Batteries. J. Membr. Sci..

[11] Son T.Y., Ko T.H., Vijayakumar V., Kim K., Nam S.Y. (2020). Anion Exchange Composite Membranes Composed of Poly (Phenylene Oxide) Containing Quaternary Ammonium and Polyethylene Support for Alkaline Anion Exchange Membrane Fuel Cell Applications. Solid State Ion..

[12] Son T.Y., Kim T., Nam S.Y. (2020). Crosslinked Pore-Filling Anion Exchange Membrane using the Cylindrical Centrifugal Force for Anion Exchange Membrane Fuel Cell System. Polymers.

[13] Son T.Y., Kim D.J., Vijayakumar V., Kim K., Kim D.S., Nam S.Y. (2020). Anion Exchange Membrane using Poly (Ether Ether Ketone) Containing Imidazolium for Anion Exchange Membrane Fuel Cell (AEMFC). J. Ind. Eng. Chem..

[14] Li N., Cui Z., Zhang S., Li S., Zhang F. (2007). Preparation and Evaluation of a Proton Exchange Membrane Based on Oxidation and Water Stable Sulfonated Polyimides. J. Power Sources.

[15] Lee J.Y., Lim D., Chae J.E., Choi J., Kim B.H., Lee S.Y., Yoon C.W., Nam S.Y., Jang J.H., Henkensmeier D. (2016). Base Tolerant Polybenzimidazolium Hydroxide Membranes for Solid Alkaline-Exchange Membrane Fuel Cells. J. Membr. Sci..

[16] Li S., Zhu X., Liu D., Sun F. (2018). A Highly Durable Long Side-Chain Polybenzimidazole Anion Exchange Membrane for AEMFC. J. Membr. Sci..

[17] Son T.Y., Choi D.H., Park C.H., Nam S.Y. (2017). Preparation and Electrochemical Characterization of Membranes using Submicron Sized Particles with High Ion Exchange Capacity for Electro-Adsorptive Deionization. J. Nanosci. Nanotechnol..

[18] Mohanty A.D., Ryu C.Y., Kim Y.S., Bae C. (2015). Stable Elastomeric Anion Exchange Membranes Based on Quaternary Ammonium-Tethered Polystyrene-b-Poly (Ethylene-Co-Butylene)-b-Polystyrene Triblock Copolymers. Macromolecules.

[19] Jeon J.Y., Park S., Han J., Maurya S., Mohanty A.D., Tian D., Saikia N., Hickner M.A., Ryu C.Y., Tuckerman M.E. (2019). Synthesis of Aromatic Anion Exchange Membranes by Friedel–Crafts Bromoalkylation and Cross-Linking of Polystyrene Block Copolymers. Macromolecules.

[20] Khataee A., Pan D., Olsson J.S., Jannasch P., Lindström R.W. (2021). Asymmetric Cycling of Vanadium Redox Flow Batteries with a Poly(Arylene Piperidinium)-Based Anion Exchange Membrane. J. Power Sources.

[21] Chen N., Lee Y.M. (2021). Anion Exchange Polyelectrolytes for Membranes and Ionomers. Prog. Polym. Sci..

[22] Vincent I., Bessarabov D. (2018). Low Cost Hydrogen Production by Anion Exchange Membrane Electrolysis: A Review. Renew. Sustain. Energy Rev..

[23] Chen D., Hickner M.A., Agar E., Kumbur E.C. (2013). Selective Anion Exchange Membranes for High Coulombic Efficiency Vanadium Redox Flow Batteries. Electrochem. Commun..

[24] Yun S., Parrondo J., Ramani V. (2014). Derivatized Cardo-Polyetherketone Anion Exchange Membranes for all-Vanadium Redox Flow Batteries. J. Mater. Chem. A.

[25] Zhang D., Yan X., He G., Zhang L., Liu X., Zhang F., Hu M., Dai Y., Peng S. (2015). An Integrally Thin Skinned Asymmetric Architecture Design for Advanced Anion Exchange Membranes for Vanadium Flow Batteries. J. Mater. Chem. A.

[26] Huang K., Li X., Liu S., Tan N., Chen L. (2008). Research Progress of Vanadium Redox Flow Battery for Energy Storage in China. Renew. Energy.

[27] Mohammadi T., Kazacos M.S. (1996). Modification of Anion-Exchange Membranes for Vanadium Redox Flow Battery Applications. J. Power Sources.

[28] Mai Z., Zhang H., Li X., Xiao S., Zhang H. (2011). Nafion/polyvinylidene Fluoride Blend Membranes with Improved Ion Selectivity for Vanadium Redox Flow Battery Application. J. Power Sources.

[29] Im K.S., Lee J.W., Jang J.Y., Nam S.Y. (2019). Hydrophilic Coating and Characterization of PVDF Membrane with Flower Type Cross-section made from Thermally Induced Phase Separation. Membr. J..

[30] Cho H., Atanasov V., Krieg H.M., Kerres J.A. (2020). Novel Anion Exchange Membrane Based on Poly (Pentafluorostyrene) Substituted with Mercaptotetrazole Pendant Groups and its Blend with Polybenzimidazole for Vanadium Redox Flow Battery Applications. Polymers.

[31] Lim H., Lee B., Yun D., Al Munsur A.Z., Chae J.E., Lee S.Y., Kim H.J., Nam S.Y., Park C.H., Kim T. (2018). Poly (2,6-dimethyl-1,4-phenylene oxide)s with various head groups: Effect of head groups on the properties of anion exchange membranes. ACS. Appl. Mater. Interfaces.

[32] Chen D., Hickner M.A., Agar E., Kumbur E.C. (2013). Optimizing Membrane Thickness for Vanadium Redox Flow Batteries. J. Membr. Sci..

[33] Huang X., Wang W., Liu Y., Wang H., Zhang Z., Fan W., Li L. (2015). Treatment of Oily Waste Water by PVP Grafted PVDF Ultrafiltration Membranes. Chem. Eng. J..

[34] Brewis D., Mathieson I., Sutherland I., Cayless R., Dahm R. (1996). Pretreatment of Poly (Vinyl Fluoride) and Poly (Vinylidene Fluoride) with Potassium Hydroxide. Int. J. Adhes. Adhes..

[35] Elgammal R.A., Tang Z., Sun C., Lawton J., Zawodzinski T.A. (2017). Species Uptake and Mass Transport in Membranes for Vanadium Redox Flow Batteries. Electrochim. Acta.

[36] Wang T., Jeon J.Y., Han J., Kim J.H., Bae C., Kim S. (2020). Poly(Terphenylene) Anion Exchange Membranes with High Conductivity and Low Vanadium Permeability for Vanadium Redox Flow Batteries (VRFBs). J. Membr. Sci..

[37] Xi J., Wu Z., Qiu X., Chen L. (2007). Nafion/SiO_2_ Hybrid Membrane for Vanadium Redox Flow Battery. J. Power Sources.

[38] Lu S., Wu C., Liang D., Tan Q., Xiang Y. (2014). Layer-by-Layer Self-Assembly of Nafion–[CS–PWA] Composite Membranes with Suppressed Vanadium Ion Crossover for Vanadium Redox Flow Battery Applications. RSC Adv..

